# Public knoweldge of sepsis in Saudi Arabia: A cross-sectional study

**DOI:** 10.1097/MD.0000000000042643

**Published:** 2025-05-30

**Authors:** Kadejh Abdulrahman Bashekah, Alla Hussain Felemban, Lubna Abdulrahman Hafiz, Abdulrahman Mauafaq Aljifri, Dalal Nasser Gaith Alsharif, Abdulaziz Ahmad Albarakati, Hind Mauafaq Aljifri, Sarah Mauafaq Aljifri, Hind Abdullah Ebrahim Abdullah, Hanan Ali Zurban, Mashaer Omar Fallatah, Salma Abdulkarim Alkhoutani, Saeed Ali Alzahrani

**Affiliations:** a Endocrine and Diabetes Center, Saudi Ministry of Health, Jeddah, Saudi Arabia; b King Faisal Specialist Hospital, Jeddah, Saudi Arabia; c Department of Family Medicine, Faculty of Medicine, Fakeeh College of Medical Sciences, Jeddah, Saudi Arabia; d Emergency Department, National Guard Hospital, Jeddah, Saudi Arabia; e Faculty of Medicine, Fakeeh College of Medical Sciences, Jeddah, Saudi Arabia; f Faculty of Medicine, King Abdulaziz University, Jeddah, Saudi Arabia; g King Abdulaziz Hospital, Saudi Ministry of Health, Jeddah, Saudi Arabia.

**Keywords:** awareness, general public, knowledge, Saudi Arabia, spesis

## Abstract

Sepsis is a life-threatening complication of the body’s response to an infection that can result in the malfunction of organs and tissues. The majority of sepsis cases are managed in the community. This study aimed to evaluate the general public’s awareness of sepsis in Saudi Arabia. A cross-sectional online survey was undertaken between November 2023 and June 2024 in Saudi Arabia to investigate public knowledge of sepsis in Saudi Arabia. A previously developed questionnaire was used in this study. Binary logistic regression analysis was utilized to determine the variables that influence sepsis awareness. A total of 1010 participants were involved in this study. The median knowledge score the study participants was 4.00 (IQR: 2.00–7.00); which demonstrates weak level of knowledge. Individuals aged 61 years and over had significantly lower odds of having higher sepsis knowledge compared to younger adults aged 18 to 23 years (OR = 0.34; 95% CI 0.14–0.79, *P* = .012). Participants holding a diploma (OR = 0.46; 95% CI 0.24–0.87, *P* = .017) showed lower odds compared to those with a high school education or lower. Married (OR = 2.14; 95% CI 1.51–3.01, *P* < .001) and divorced individuals (OR = 2.82; 95% CI 1.83–4.35, *P* < .001) demonstrated higher odds of possessing greater sepsis knowledge compared to single individuals. Participants earning 2500 to 5000 Saudi Riyal (SAR) (OR = 2.05; 95% CI 1.22–3.44, *P* = .006) and 7500 SAR and above (OR = 1.63; 95% CI 1.11–2.41, *P* = .013) showed higher odds compared to those earning <2500 SAR. Employment in the healthcare sector (OR = 4.13; 95% CI 2.41–7.06, *P* < .001) and being a medical student (OR = 4.99; 95% CI 1.09–22.90, *P* = .039) were also associated with significantly higher odds of having greater sepsis knowledge. Sepsis, its symptoms, and its risk factors are not well understood by the general public, according to our research. These findings emphasize the necessity for sepsis education to raise public awareness. Awareness can help people with this life-threatening disorder get diagnosed earlier and have favorable outcomes.

## 1. Introduction

Sepsis is a life-threatening complication of the body’s response to an infection that can result in the malfunction of organs and tissues.^[1]^ In the United States and Canada, sepsis was the leading cause of in-hospital fatality and accounted for over 20% of all global in-hospital mortality.^[2–4]^ The severity of sepsis results in long-term neurocognitive, psychological, somatic, and medical complications, as well as billions of dollars in hospitalization management costs.^[4–7]^ This has been estimated to have deteriorated as a result of the introduction of severe acute respiratory syndrome coronavirus-2.^[8,9]^ Critically ill individuals with severe coronavirus disease-2019 (COVID-19) are diagnosed with sepsis.^[10,11]^ In addition, the long-term morbidities that have been reported among a significant number of COVID-19 survivors are comparable to those that have been previously documented in sepsis survivors.^[9]^ The timely provision of treatment and the improvement of long-term patient outcomes are contingent upon the early identification of the signs and symptoms of sepsis.^[12,13]^ Among the evidence-based clinical guidelines and practices bundles that have been key features of hospital-based strategies for the improvement of timely diagnosis and response to sepsis are the Surviving Sepsis International Guidelines for Management of Sepsis and Septic Shock. These guidelines provide recommendations of care for adult patients and children with sepsis or septic shock.^[14,15]^ Nevertheless, the majority of sepsis cases are managed in the community.^[16]^ Consequently, it is essential to enhance the public’s and outpatients’ understanding of sepsis in order to facilitate the development process.^[12,17,18]^

In 2021, a study by Humoodi et al in Saudi Arabia reported that 4.9% of patients admitted to pediatric intensive care unit were diagnosed with sepsis.^[19]^ Another recent national study conducted in 440 hospitals in Saudi Arabia reported that 12.8% of the patients had confirmed sepsis diagnosis in 2023.^[20]^ In 2022, a scoping review was conducted to identify and map the literature on sepsis awareness, general knowledge, and information-seeking behaviors among healthcare professionals, patients, and the public.^[21]^ The general public’s awareness of the term “sepsis” fluctuates from 4% in France to 88% in Germany.^[22,23]^ Despite the fact that awareness has improved incrementally in recent studies, less than half of the participants in the majority of public-focused studies reported that they were personally aware of sepsis.^[21]^ Nevertheless, this may be attributed to the increasing impact of awareness campaigns conducted by governments through education, such as the Centers for Disease Control and Prevention, and non-governmental organizations, such as the Global Sepsis Alliance.^[24]^ The aim of this study is to evaluate the general public’s awareness of sepsis in Saudi Arabia.

## 2. Methods

### 2.1. Study design and settings

A cross-sectional online survey was conducted between November 2023 and June 2024 in Saudi Arabia to investigate public knowledge of sepsis in Saudi Arabia.

### 2.2. Sampling procedure

The sample for this research was selected through a method referred to as convenience sampling. This type of sampling falls within the category of non-probability sampling. The use of convenience sampling technique enhances the requitement of study participants within considerable time and efforts. The present study encompassed individuals who satisfy the predetermined criteria for inclusion and express their willingness to partake in the research. At the commencement of the questionnaire, participants were presented with an informed consent form, affording them the opportunity to either proceed with their involvement in the study or opt to withdraw from it. In order to enhance patients’ understanding of the significance of their involvement, the research objectives were clearly presented in their entirety. The invitation letter for the study provided a detailed overview of the inclusion criteria.

### 2.3. Study population and recruitment

The population for this study comprised of individuals who are at least 18 years old and are residents of Saudi Arabia, belonging to the general community. There are no restrictions based on age or gender. The survey hyperlink was disseminated across several social media platforms, including Facebook, Snapchat, WhatsApp, and Twitter, with the aim of fostering increased engagement and involvement.

### 2.4. Study tool

A previously developed questionnaire was used in this study. They first created a preliminary list of questions from published articles identified in a scoping review of publications examining public awareness and knowledge of sepsis related to knowledge of sepsis,^[21]^ see Appendix 1, Supplemental Digital Content, https://links.lww.com/MD/P54. In addition, participants’ demographic characteristics (age, gender, nationality, education, monthly income category, employment status, and marital status) were collected from the study participants. Knowledge of sepsis was examined in terms of its definition, signs and symptoms, mortality and risk factors, and prevention. Based on the identification of response options to each knowledge question, we computed composite knowledge scores for each respondent. Response options that were correctly selected were coded as 1, while those that were incorrect or marked as “don’t know” were coded as 0. The maximum knowledge score was 26, the higher the score the higher the level of knowledge concerning sepsis.

### 2.5. Piloting of the questionnaire tool

The questionnaire instrument was evaluated and validated by medical professionals affiliated with the Saudi Ministry of Health. The participants were queried regarding the clarity, comprehensibility, and face validity of the questions, in addition to any difficulties encountered in understanding them. Furthermore, the participants were requested to provide feedback regarding any inquiries that they perceived as unpleasant. Furthermore, a preliminary investigation was conducted using a limited sample from the target audience to assess their comprehension of the survey instrument prior to its extensive implementation.

### 2.6. Survey translation

The forward–backward translation technique was implemented in this study. Two independent reviewers translated the questionnaire independently. The translation was checked by a third reviewer by comparing the translated draft with the original tool.

### 2.7. Sample size

The minimum required sample size was 385 individuals using a 95% confidence interval, a 0.5 standard deviation, and a 5% margin of error.

### 2.8. Ethical approval

This study was reviewed by the Institutional Review Board at Ministry of Health, Jeddah, Saudi Arabia (IRB approval number A01835). Participants were informed that completing the questionnaire is considered an informed consent for participation.

### 2.9. Statistical analysis

**Figure 1.**
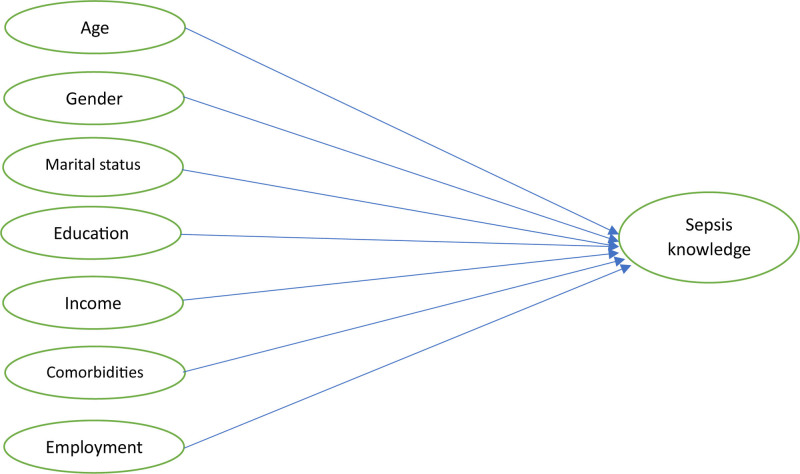
The study conceptual framework.

Using SPSS version 27, this study’s data were analyzed. Histogram and normality metrics were used to examine the normality of public awareness score. Based on the normality of the data, the awareness score was presented as median and interquartile range as the knowledge score was not normally distributed, which was demonstrated using the histogram and other normality measures (kurtosis value of 1.245 and skewness value of 1.125). In a binary logistic regression analysis, the median awareness score (which is equal to 4.00) of the participants was utilized as the dummy variable to determine the variables that influence sepsis awareness (Fig. 1). To determine statistical significance, a two-sided *P*-value <.05 was utilized.

## 3. Results

Table 1 below presents participants’ demographic characteristics. A total of 1010 participants were involved in this study. The majority of participants were female (82.4%) and married (73.7%). Most were aged between 41 to 50 years (28.6%), followed closely by those aged 51 to 60 years (28.3%). In terms of education, the highest percentage of participants had a bachelor degree (55.2%). A significant portion of participants reported a monthly income of 7500 Saudi Riyal (SAR) and above (64.9%). Employment status was diverse, with the largest group being unemployed (32.2%), followed by retired individuals (28.3%). Additionally, 42.9% of participants reported having a chronic disease.

**Table 1:** Participants’ demographic characteristics.

Variable	Frequency	Percentage
*Gender*		
Females	832	82.4%
*Age*		
18–23 years	31	3.1%
24–30 years	63	6.2%
31–40 years	142	14.1%
41–50 years	289	28.6%
51–60 years	286	28.3%
61 year and over	199	19.7%
*Level of education*		
High school or lower	176	17.4%
Diploma	99	9.8%
Bachelor	558	55.2%
Higher education	177	17.5%
*Marital status*		
Single	127	12.6%
Married	744	73.7%
Divorced	84	8.3%
Widowed	55	5.4%
*Monthly income level*		
<2500 SAR	121	12.0%
2500–5000 SAR	119	11.8%
5000–7500 SAR	115	11.4%
7500 SAR and above	655	64.9%
*Employment status*		
Unemployed	325	32.2%
Retired	286	28.3%
Work outside healthcare sector	262	25.9%
Work in healthcare sector	111	11.0%
Medical student	13	1.3%
None medical student	13	1.3%
Do you have any chronic disease? (Yes)	433	42.9%

### 3.1. Sepsis knowledge profile

The median knowledge scores the study participants was 4.00 (IQR: 2.00–7.00); which demonstrates weak level of knowledge. Table 2 below presents participants’ responses to questions that examined their knowledge of sepsis. For the definition of sepsis, 31.2% correctly identified it as the body’s extreme response to an infection, and 67.9% correctly stated that sepsis is not contagious. A total of 12.5%, 24.7%, and 25.2%, respectively correctly identified that infection, inflammation, and the body’s extreme response to an infection are phrases that describe sepsis. In terms of signs and symptoms, 37.0% knew that sepsis involves a combination of symptoms rather than a single indicator. Fever was the most commonly reported symptom or sign of sepsis (37.9%). The least commonly reported symptom or sign of sepsis was slurred speech or confusion (3.5%).

**Table 2:** Participants’ responses to questions that examined their knowledge of sepsis.

Domain	Question	Percentage of participants
Definition	*Which of the following statements about sepsis is true?*	
	Sepsis is a severe allergic reaction	14.9%
	Sepsis is a seizure involving violent muscle contractions	2.7%
	Sepsis is the body’s extreme response to an infection	31.2%
	I don’t know	51.3%
	*Select the word(s) or phrase(s) that describe sepsis.*	
	Allergic reaction	12.4%
	Poisoning by eating contaminated food	16.4%
	Infection	12.5%
	Inflammation	24.7%
	The body’s extreme response to an infection	25.2%
	None of the above responses describe sepsis	1.6%
	I don’t know	37.5%
	*Sepsis is contagious.*	
	True	5.1%
	False	67.9%
	I don’t know	26.9%
Signs & symptoms	*Which of the following statements about symptoms of sepsis is true?*	
	Weakness or numbness on one side of the body is a common symptom of sepsis	4.1%
	An infected wound with bloody pus is always a symptom of sepsis	9.2%
	Sepsis is associated with a combination of symptoms (no single symptom indicates sepsis)	37.0%
	I don’t know	49.7%
	*Which of the following, if any, are common symptoms or signs of sepsis*	
	Fever	37.9%
	Feeling extremely ill (like you are going to die)	24.9%
	Skin blotchy or discolored	18.6%
	Fast heart rate	18.2%
	Fast breathing/severe breathlessness	17.0%
	Extreme shivering or muscle pain	11.9%
	Infection	8.5%
	Passing no urine all day	4.5%
	Slurred speech or confusion	3.5%
	Indigestion	4.1%
	Weakness or numbness on one side of the body	3.3%
	Others	2.8
	I don’t know	42.5%
Mortality & risk factors	*Sepsis is one of the leading cause of death worldwide.*	
	True	20.3%
	False	27.3%
	I don’t know	52.4%
	*Roughly what percentage of deaths around the world are due to sepsis each year?*	
	5%	6.3%
	15%	7.9%
	20%	5.6%
	30%	4.8%
	I don’t know	75.3%
	*Which of the following factors are associated with a higher risk of a person developing sepsis?*	
	preexisting medical conditions (e.g., diabetes)	37.0%
	Age	11.5%
	Living in a shared housing facility (e.g., nursing home)	7.2%
	Race/ethnicity	3.4%
	Sex	2.5%
	Income level	2.6%
	Education level	2.4%
	I don’t know	53.3%
Prevention	*Which of the following actions, if any, can help prevent or lower your risk of developing sepsis?*	
	Treating infections	35.0%
	Drinking lots of fluids	19.0%
	Eating a balanced diet	16.7%
	Personal hygiene (keeping your body clean)	15.0%
	Hand washing	12.2%
	Keeping vaccinations up to date (e.g., seasonal influenza (flu) shot, SARS-CoV-2 (COVID-19) shot)	11.3%
	Getting 8 hours of sleep a night	7.3%
	None, sepsis cannot be prevented	6.7%
	I don’t know	40.8%

SARS-Cov-2 = severe acute respiratory syndrome coronavirus-2.

Regarding mortality and risk factors, 20.3% correctly answered that sepsis is one of the leading causes of death worldwide. Around 5.6% correctly identified that the percentage of deaths around the world are due to sepsis each year is around 20.0%. The most commonly reported factors associated with a higher risk of a person developing sepsis was preexisting medical conditions (e.g., diabetes) (37.0%). Concerning prevention, around 35.0% of the participants reported that treating infections can help prevent or lower your risk of developing sepsis.

### 3.2. Predictors of higher level of sepsis knowledge

Table 3 below presents the findings of logistic regression analysis. Individuals aged 61 years and over had significantly lower odds of having higher sepsis knowledge compared to younger adults aged 18 to 23 years (OR = 0.34; 95% CI 0.14–0.79, *P* = .012). Education level also influenced sepsis knowledge, with those holding a diploma (OR = 0.46; 95% CI 0.24–0.87, *P* = .017) having lower odds compared to those with a high school education or lower. Marital status was a significant predictor, with married (OR = 2.14; 95% CI 1.51–3.01, *P* < .001) and divorced individuals (OR = 2.82; 95% CI 1.83–4.35, *P* < .001) demonstrating higher odds of possessing greater sepsis knowledge compared to single individuals. Income level played a role, with those earning 2500 to 5000 SAR (OR = 2.05; 95% CI 1.22–3.44, *P* = .006) and 7500 SAR, and above (OR = 1.63; 95% CI 1.11–2.41, *P* = .013) having higher odds compared to those earning <2500 SAR. Employment in the healthcare sector (OR = 4.13; 95% CI 2.41–7.06, *P* < .001) and being a medical student (OR = 4.99; 95% CI 1.09–22.90, *P* = .039) were also associated with significantly higher odds of having greater sepsis knowledge compared to retired individuals.

**Table 3:** Predictors of higher level of sepsis knowledge.

Variable	Odds ratio of having higher knowledge (95% confidence interval)	*P*-value
*Gender*		
Females (Reference group)	1.00	
Males	0.95 (0.69–1.32)	.756
*Age*		
18–23 years (Reference group)	1.00	
24–30 years	0.87 (0.33–2.30)	.778
31–40 years	0.55 (0.23–1.32)	.180
41–50 years	0.44 (0.19–1.01)	.053
51–60 years	0.47 (0.20–1.08)	.0.75
61 year and over	0.34 (0.14–0.79)	.012*
*Level of education*		
High school or lower (Reference group)	1.00	
Bachelor	0.76 (0.51–1.12)	.169
Higher education	0.48 (0.27–1.83)	.051
Diploma	0.46 (0.24–0.87)	.017*
*Marital status*		
Single (Reference group)	1.00	
Married	2.14 (1.51–3.01)	<.001
Divorced	2.82 (1.83–4.35)	<.001
Widowed	1.50 (0.91–2.46)	.109
*Monthly income level*		
<2500 SAR (Reference group)	1.00	
2500–5000 SAR	2.05 (1.22–3.44)	.006**
5000–7500 SAR	1.41 (0.84–2.35)	.192
7500 SAR and above	1.63 (1.11–2.41)	.013*
*Employment status*		
Retired (Reference group)	1.00	
Unemployed	0.99 (0.72–1.37)	.972
Work in healthcare sector	4.13 (2.41–7.06)	<.001
Medical student	4.99 (1.09–22.90)	.039*
Work outside healthcare sector	1.20 (0.85–1.68)	.299
None medical student	1.06 (0.35–3.23)	.921
*Do you have any chronic disease?*		
No (Reference group)	1.00	
Yes	0.99 (0.77–1.27)	.919

SAR: Saudi Riyal.

* *P* < .01.

** *P* < .05.

## 4. Discussion

Sepsis is a life-threatening condition caused by the body’s response to an infection, which leads to widespread inflammation, tissue damage, organ failure, and, potentially, death. Despite advances in medical care, sepsis continues to be a significant global health challenge, resulting in substantial morbidity and mortality. Our study revealed that Saudi Arabian participants showed a moderate to low level of knowledge about sepsis.

We found that approximately 31.2% of participants correctly identified sepsis as the body’s extreme response to an infection. This percentage is similar to studies conducted in Korea,^[25]^ as well as other studies with participants from various European countries and the USA,^[23]^ which also reported comparable levels of awareness. However, a Canadian study showed a significantly higher percentage, with around 65% of participants correctly identifying sepsis.^[26]^ This difference may be attributed to variations in public health education across these populations. These variations underscore the need for educational programs to enhance sepsis knowledge in regions with lower awareness levels.

Our study also revealed low percentages of participants correctly identifying inflammation, infection, and the body’s severe reaction to infection as terms that describe sepsis, which were 12.5%, 24.7%, and 25.2%, respectively. These low percentages emphasize the urgent need to raise awareness about sepsis among the general population. In contrast, a Canadian study reported significantly higher percentages of accurate identification.^[26]^ This discrepancy highlights the effectiveness of Canadian efforts to raise public knowledge and understanding of sepsis and reinforces the necessity for similar programs in our area to enhance sepsis awareness and understanding among the general public.

A complex upset of the delicately balanced immunological balance between inflammation and anti-inflammation is part of the pathogenesis of sepsis. The result of this imbalance is an immune response that is uncontrollably activated by both pro- and anti-inflammatory pathways. The body’s immune system is activated in response to an infectious pathogen, causing the release of several cytokines and other mediators.^[27]^

Regarding signs and symptoms, 37.0% of participants recognized that sepsis involves a combination of symptoms rather than a single indicator. Fever was reported as the most common symptom or sign of sepsis (37.9%), while slurred speech or confusion was reported the least frequently (3.5%). Another study in Saudi Arabia reported that heartbeat (28.6%), difficulty in breathing (20.7%), lack of concentration (13.3%), and fever (31.5%) as the most common septic symptoms.^[28]^ These findings align with a study conducted in Germany in 2018, which assessed knowledge and awareness of sepsis among the public. In that study, fever was the most commonly reported sign (73%), while low blood pressure and diarrhea were the least reported (19.3%) for both.^[22]^ In our study on mortality and risk factors, 20.3% of participants correctly identified sepsis as one of the leading causes of death worldwide. Additionally, 5.6% of participants accurately recognized that around 20.0% of global deaths each year are attributable to sepsis. Several studies demonstrated that patients with sepsis commonly complained in hospitals and primary cares of severe pain and extreme malaise. Other signs included fever, the patient falling or collapsing, high respiratory rate and extreme muscle weakness.^[29–32]^

Among the commonly reported risk factors for sepsis were preexisting medical conditions like diabetes, which was identified by 37.0% of participants. Another study conducted in the western region of Saudi Arabia showed that 19.7% of participants recognized diabetes as a risk factor for sepsis.^[28]^ Other risk factors reported by the previous Saudi study included burns and injuries (19.2%), intubation and catheterization (26.1%), infection (39.4%), severe illness (38.9%), and low immunity (75.9%).^[28]^ These findings demonstrate consistent awareness among participants regarding the impact of diabetes and other conditions on the risk of developing sepsis. It highlights the importance of targeted educational efforts to enhance sepsis knowledge across different regions, particularly concerning risk factors and mortality rates. In a Canadian study, age was treated as continuous data and found to be a significant factor influencing public knowledge (OR = 1.00, *P* = .005).^[26]^ However, our findings differed from this. We observed that individuals aged 61 years and over had significantly lower odds of possessing higher sepsis knowledge compared to younger adults aged 18 to 23 years (OR = 0.34; 95% CI 0.14–0.79, *P* = .012). This discrepancy may be attributed to older individuals having limited access to sepsis-related information in our study.

Marital status emerged as a significant predictor in our study. Married individuals (OR = 2.14; 95% CI 1.51–3.01, *P* < .001) and divorced individuals (OR = 2.82; 95% CI 1.83–4.35, *P* < .001) demonstrated higher odds of possessing greater sepsis knowledge compared to single individuals. This finding suggests that married individuals may have more opportunities for health-related discussions and information sharing, potentially leading to greater awareness and understanding of health conditions like sepsis.

Income level also played a role in our study. Participants earning 2500 to 5000 SAR (OR = 2.05; 95% CI 1.22–3.44, *P* = .006) and 7500 SAR and above (OR = 1.63; 95% CI 1.11–2.41, *P* = .013) had higher odds of possessing greater sepsis knowledge compared to those earning <2500 SAR. However, these findings differ from a study conducted in Canada, which found that income had no significant impact on sepsis awareness (β = 0.328, *P* = .24). This inconsistency may be attributed to differences in educational programs between the 2 countries. In our study, we found that employment in the healthcare sector (OR = 4.13; 95% CI 2.41–7.06, *P* < .001) and being a medical student (OR = 4.99; 95% CI 1.09–22.90, *P* = .039) were associated with significantly higher odds of having greater sepsis knowledge compared to retired individuals. These findings align with a study by Jeanna Parsons Leigh et al, 2022, which highlighted that working in healthcare is a strong predictor of knowledge among the public in Canada (β = 2.756, *P* = .001).^[26]^ Moreover, these findings were consistent with the findings of a previous study in Saudi Arabia which reported that students and those who work in the healthcare sector showed significantly higher level of awareness compared to others.^[28]^ This similarity emphasizes the importance of healthcare experience in sepsis education and awareness.

Based on our study findings, public awareness campaigns should target older adults and those with low education level. Awareness campaigns should be launched in social media and other public organizations. This could be done in collaboration with healthcare providers. Awareness campaigns should inform the participants on signs, symptoms, and risk factors of sepsis. Besides, they should inform them on the necessity for early detection and intervention in this study of conditions.

However, it is important to acknowledge the limitations of our study. One limitation relates to the study design, as the accuracy of the data relies on participants’ ability to accurately recall information, which can introduce reporting bias. Another limitation is that our study only provides a snapshot of public knowledge at a specific point in time, without considering potential changes in knowledge over time. Additionally, the survey was distributed online using convenience sampling technique, which may have limited participation from elderly individuals and those less familiar with the internet and technology and decrease the generalizability of the study findings. Future studies utilizing stratified sampling technique are warranted. Moreover, this type of self-administered studies is prone to reporting bias. Therefore, our study findings should be interpreted carefully.

## 5. Conclusion

Our findings showed that there is a low level of knowledge among the public about sepsis, its signs and symptoms, and its risk factors. These findings highlight the need to enhance public awareness through educational activities, which aim to improve public knowledge about sepsis. Increased awareness can lead to earlier recognition and potentially better outcomes for individuals affected by this life-threatening condition. Further studies should be conducted to identify the knowledge gap and to develop sepsis awareness programs targeted at specific populations, such as the general public, patients, and healthcare providers.

## Author contributions

**Conceptualization:** Kadejh Abdulrahman Bashekah.

**Data curation:** Kadejh Abdulrahman Bashekah, Mashaer Omar Fallatah, Salma Abdulkarim Alkhoutani, Saeed Ali Alzahrani.

**Formal analysis:** Kadejh Abdulrahman Bashekah.

**Funding acquisition:** Kadejh Abdulrahman Bashekah.

**Investigation:** Kadejh Abdulrahman Bashekah, Alla Hussain Felemban, Lubna Abdulrahman Hafiz, Abdulrahman Mauafaq Aljifri, Dalal Nasser Gaith Alsharif, Abdulaziz Ahmad Albarakati, Hind Mauafaq Aljifri, Sarah Mauafaq Aljifri, Hind Abdullah Ebrahim Abdullah, Hanan Ali Zurban, Mashaer Omar Fallatah, Salma Abdulkarim Alkhoutani, Saeed Ali Alzahrani.

**Methodology:** Kadejh Abdulrahman Bashekah.

**Project administration:** Kadejh Abdulrahman Bashekah.

**Resources:** Kadejh Abdulrahman Bashekah, Alla Hussain Felemban, Lubna Abdulrahman Hafiz, Abdulrahman Mauafaq Aljifri, Dalal Nasser Gaith Alsharif, Abdulaziz Ahmad Albarakati, Hind Mauafaq Aljifri, Sarah Mauafaq Aljifri, Hind Abdullah Ebrahim Abdullah, Hanan Ali Zurban, Mashaer Omar Fallatah, Salma Abdulkarim Alkhoutani, Saeed Ali Alzahrani.

**Software:** Kadejh Abdulrahman Bashekah.

**Supervision:** Kadejh Abdulrahman Bashekah.

**Validation:** Kadejh Abdulrahman Bashekah.

**Visualization:** Kadejh Abdulrahman Bashekah.

**Writing – original draft:** Kadejh Abdulrahman Bashekah.

**Writing – review & editing:** Kadejh Abdulrahman Bashekah, Alla Hussain Felemban, Lubna Abdulrahman Hafiz, Abdulrahman Mauafaq Aljifri, Dalal Nasser Gaith Alsharif, Abdulaziz Ahmad Albarakati, Hind Mauafaq Aljifri, Sarah Mauafaq Aljifri, Hind Abdullah Ebrahim Abdullah, Hanan Ali Zurban, Mashaer Omar Fallatah, Salma Abdulkarim Alkhoutani, Saeed Ali Alzahrani.

## Supplementary Material


