# Supplementary material for: Public knoweldge of sepsis in Saudi Arabia: A cross-sectional study
Source: Medicine (Baltimore). 2025 May 30;104(22):e42643. doi: 10.1097/MD.0000000000042643 (PMC12129535; doi:10.1097/MD.0000000000042643)
Supplement: Supplementary file 1 [file medi-104-e42643-s001.pdf]

Questionnaire tool

السلام عليكم ورحمة الله وبركاته
نحن مجموعة من الاطباء بجده هدفنا دراسة مدى معرفة عامة الشعب في المملكة العربية السعودية بمرض تسمم الدم.
اذا عمرك 18 سنه أو اكثر يرجى التكرم بالمشاركة معنا في هذه الدراسة .
ليس هناك جواب صحيح أو خاطئ .سيستغرق ملء الاستبيان اقل من 5 دقائق .
مساهمتك قيمة بالنسبة لنا ، لأنها ستساعد على تحسين الرعاية الصحية.
هذا الاستبيان مجهولٌ تماماً ولن يتمكن الباحثون من التعرف عليك من الردود المقدمة .
لن يطلب منك اسمك في أي مكان في الاستبيان .
سوف نستخدم المعلومات التي تم جمعها لأغراض البحث فقط .
إكمالك للإستبيان يعتبر موافقة على المشاركة بالبحث للتواصل G.research12345@gmail.com :

الجنس:

- انثى
- ذكر

الفئة العمرية:

- 18-23سنة
- 24-30سنة
- 31-40سنة
- 41-50سنة
- 51-60سنة
- 61فما فوق

الحالة الاجتماعية:

- اعزب - عزباء
- متزوج - متزوجه
- مطلق - مطلقه
- ارمل - ارمله

المستوى التعليمي:

- دراسه ثانوية أو أقل
- الدبلوم
- بكالوريوس
- تعليم عالي

الدخل الشهري للأسره:

- أقل من 2500ريال
- 2500-5000ريال
- 5000-7500ريال
- 7500ريال او اكثر

الوضع الوظيفي:

- متقاعد
- لا اعمل
- اعمل في المجال الطبي
- اعمل خارج المجال الطبي
- طالب جامعي في الكليات الصحية
- طالب جامعي في كليات غير صحية

هل تعاني من أي امراض مزمنة مثل امراض القلب أو السكري أو الضغط أو ارتفاع مستوى الدهنيات أو غيرها؟

- لا
- نعم

أي من العبارات التالية حول تسمم الدم" خمج الدم "صحيحة؟

- تسمم الدم هو رد فعل تحسسي شديد
- تسمم الدم هو نوبة تنطوي على تقلصات عضلية عنيفة
- تسمم الدم هو استجابة الجسم الشديدة للعدوى
- لا أعرف

أي من العبارات التالية حول أعراض تسمم الدم صحيحة؟

- ◯ يعد الضعف أو التنميل في أحد جانبي الجسم من الأعراض الشائعة لتسمم الدم
- ◯ دائمًا ما يكون الجرح المصاب بالقيح الدموي من أعراض تسمم الدم
- ◯ يرتبط تسمم الدم بمجموعة من الأعراض) لا يوجد عرض واحد يشير إلى تسمم الدم (
- ◯ لا أعرف

حدد الكلمة) الكلمات (أوالعبارات التي تصف أعراض تسمم الدم.

- ▢ رد فعل تحسسي
- ▢ التسمم عن طريق تناول الأطعمة الملوثة
- ▢ عدوى
- ▢ التهاب
- ▢ استجابة الجسم الشديدة للعدوى
- ▢ لا تصف أي من الاجابات المذكورة أعلاه تسمم الدم
- ▢ لا أعرف

أي مما يلي، إن وجد، يعد من الأعراض أو علامات تسمم الدم الشائعة

- حمى
- عدوى
- الشعور بالمرض الشديد) وكأنك ستموت (
- ألم في الكتف الأيسر
- تلعثم الكلام أو الارتباك
- عسر الهضم
- معدل ضربات القلب سريع
- عدم التبول طوال اليوم
- التنفس السريع / ضيق التنفس الشديد
- ضعف أو تنميل في جانب واحد من الجسم
- الارتعاش الشديد أو آلام العضلات
- ظهور بقع على الجلد أو تغير لونه
- لا يعد أي مما سبق أعراضًا أو علامات شائعة لتسمم الدم
- لا أعرف

تسمم الدم معدي:

- نعم
- لا
- لا اعرف

تسمم الدم هو السبب الرئيسي للوفاة في جميع أنحاء العالم مقارنة بجميع الحالات الطبية الأخرى:

- نعم
- لا
- لا اعرف

ما هي النسبة المئوية تقريبًا للوفيات حول العالم الناجمة عن تسمم الدم كل عام؟

- 5%
- 15%
- 20%
- 30%
- لا أعرف

أي من العوامل التالية يرتبط بزيادة خطر إصابة الشخص بتسمم الدم ؟

- عمر
- مستوى الدخل
- الجنس
- مستوى التعليم
- العِرق
- العيش في منشأة سكنية مشتركة) مثل دار رعاية المسنين (
- الامراض و المشاكل الطبية الموجودة مسبقًا) مثل مرض السكري (
- لا أعرف

أي من الإجراءات التالية، إن وجدت، يمكن أن يساعد في منع أو تقليل خطر الإصابة بتسمم الدم ؟

- شرب الكثير من السوائل
- الحفاظ على أخذ التطعيمات بشكل مستمر) على سبيل المثال، لقاح الأنفلونزا الموسمية، لقاح-SARS-CoV-2 (COVID-19))
- الحصول على 8 ساعات من النوم ليلاً
- غسل اليدين
- تناول نظام غذائي متوازن
- النظافة الشخصية) الحفاظ على نظافة الجسم (
- علاج الالتهابات
- لا شيء، لا يمكن منع تسمم الدم
- لا أعرف
